# The Origins and Proliferation of Unfounded Comparisons Regarding the Safety of Mifepristone

**DOI:** 10.3390/biotech14020039

**Published:** 2025-05-24

**Authors:** Cameron Louttit

**Affiliations:** Charlotte Lozier Institute, Arlington, VA 22206, USA; clouttit@lozierinstitute.org

**Keywords:** abortion, communication, comparative safety, drug advertisement, FDA, mifepristone, regulation, side effects

## Abstract

As part of the substantial public discourse surrounding the distribution and use of mifepristone, which is used with misoprostol to facilitate drug-induced abortions, claims comparing the safety of this regimen to that of common pharmaceuticals have emerged and proliferated. Offered in forums ranging from social media to the Supreme Court, these claims have so gained public acceptance that they are now echoed without scrutiny and, at times, reference. Yet the simplistic slogan that “mifepristone is safer than Tylenol”, though easily disseminated, defies both an intuitive understanding of how we evaluate drug safety and our norms and regulations for doing so. Indeed, if such an assertion was attributable to the manufacturer, it would precipitate a reprimand by the FDA given the lack of specific, controlled, and head-to-head evidence rightly required for its support. To the extent that these claims persist, however, including among the outputs of medical societies, abortion centers, clinical researchers, and government officials, and to the extent that they aim to inform both individual and public decision-making, it is critical that the evidence offered for their support be thoroughly explored. Such examination reveals these claims to be wholly unfounded, offering deficient and disingenuous representations of safety for any of the drugs compared.

## 1. Introduction

Since the initial approval of the mifepristone-misoprostol abortion regimen in 2000, the fraction of drug-induced abortions in the United States has continually increased, with estimates surpassing 60% for the first time in 2023 [1]. Accompanying this trend has been a public conversation surrounding mifepristone’s distribution and safety, with a recent proliferation of comparisons between this drug regimen and common pharmaceuticals. Such comparisons are largely aimed at decreasing regulation and assuaging concerns of those contemplating abortion.

Indeed, we are now inundated with claims that the safety of this regimen compares similarly or favorably to that of many common pharmaceuticals, including acetaminophen (Tylenol), ibuprofen (Advil), aspirin, penicillin, and sildenafil (Viagra). These claims can be found not only in the mass media [2,3,4,5,6,7,8,9,10,11,12,13,14] but also in journal articles [15,16,17,18,19,20] and other academic outputs [21,22,23], legal documents [24,25,26,27,28,29], statements from government officials and their offices [30,31,32,33,34,35,36,37,38,39], and patient-facing informational material [40,41,42,43,44,45,46]. In recent years, perhaps influenced by the short form of social media, the claim has often coalesced to a concise and oft-repeated refrain that “mifepristone is safer than Tylenol”, to which varying combinations of the other listed drugs are at times appended.

The central problem with this claim is simultaneously a chief cause of its spread: its simplicity. A reductionist claim concerning the safety of a drug should ordinarily draw scrutiny, as it collapses a set of complex and vitally important considerations into a simple assertion or slogan. Instead, this particular claim and those like it have proliferated, aided in part by major medical organizations and abortion centers that should be providing full, informed consent to their patients on the risks attendant to any drug.

The scrutiny demanded by the claim that “mifepristone is safer than Tylenol” and those like it reveals what should be an obvious truth: these are entirely inappropriate comparisons that have never been, and indeed cannot be, investigated in the rigorous, scientific manner rightfully demanded of medical information. Furthermore, the evidence offered on their behalf is severely lacking, a wholly insufficient assessment of safety from which no comparison can reasonably be drawn. These are, therefore, dangerous and unfounded claims that are yet being presented to patients, policymakers, jurists, and the public as “consensus” facts on which patient decision-making should be based and regulatory action taken.

## 2. The Requirements of an Adequate Comparison

In October 2024, 18 attorneys general filed a motion for summary judgment in their lawsuit concerning U.S. Food and Drug Administration (FDA) restrictions on the distribution of mifepristone [24]. In support of this action, Attorney General William Tong of Connecticut, a participant in the suit, issued the following statement:

“Mifepristone is safer than Tylenol. We have more than 20 years of clear and conclusive scientific evidence proving that. We’re asking the judge now to cut to the chase—to strike the needless political red tape and order new rules based on science and safety” [30].

While the history of this claim will be investigated below, the statement raises a more essential question: what evidence is required to prove, clearly and conclusively, that one drug is safer than another?

Prior to adjudicating the validity of a comparison, one must establish on what grounds the comparison is being made. Drug safety, as the FDA has made clear throughout its history, is not a concept easily simplified. Across its application procedures, safety is assessed through a battery of data including that from in vitro and animal studies, previous use in humans in the U.S. or abroad, and clinical trials [47,48]. All safety information is required to be stratified amongst subgroups of demographic and/or clinical interest, including assessments “as applicable” on the effects of the drug in pediatric populations and on “reproduction and…the developing fetus” [48]. Further assessments are made across categories as disparate as the drug’s manufacturing pathway, its potential for abuse, and its interactions with other drugs. Each of these factors provides an important angle from which to assess a comprehensive picture of drug safety.

Of course, these considerations in their totality need not be represented in every communication regarding the drug. Manufacturers may justifiably desire to provide information about the drug’s safety, including through comparative claims, to medical professionals or the public via media that cannot fully encompass this complexity. It is precisely for this reason that the FDA provides regulation, guidance, and warnings related to the claims made in the public square via pharmaceutical advertisements.

A claim of this nature, according to FDA regulations, is “false, lacking in fair balance, or otherwise misleading” if it “represents or suggests that a drug is safer or more effective than another drug in some particular when it has not been demonstrated to be safer or more effective in such particular by substantial evidence or substantial clinical experience” [49]. Such evidence is described as the result of “adequate and well-controlled investigations, including clinical investigations”, and such experience as “substantial clinical experience adequately documented in medical literature or by other data” from which “qualified experts” could “fairly and responsibly” reach the same determination. To assist manufacturers in the practical implementation of these rules, legal analyses have been assembled that summarize their historical interpretation. One such resource, a practice guide for pharmaceutical advertising in the United States, notes:

“Comparative claims regarding a drug’s efficacy or safety are generally permitted if they are based on the approved indication of a drug to the same approved indication of another drug and are supported by scientifically appropriate and statistically sound data (e.g., head-to-head study, clinically relevant to patients, not false or misleading). Comparative claims should not suggest superior efficacy or safety based solely on the differences in product labeling or the results of two different studies” [50].

It is true that the claims cited in this work are made outside the purview of the manufacturers to whom such regulations are chiefly directed. Yet this guidance, beyond simply dictating these requirements, also yields translatable definitions for “false…or otherwise misleading claims”, “substantial evidence”, and “substantial clinical experience” that can and should be used to scrutinize these comparisons made in the public square. It also provides best practices for making these claims, which, as will be described, are routinely violated in comparisons regarding mifepristone. Finally, it is critical to note that these claims have been propagated by public officials, medical associations, and patient-facing groups who aim to inform policy, regulations, and patient decision-making through their assertions. These are the same realms with which the FDA is chiefly concerned as it addresses false and misleading comparisons. It is not unreasonable, therefore, to expect that statements of this gravity and influence be properly supported by adequate evidence, particularly when the statements themselves claim such evidence exists.

It is essential to state unequivocally that there has never been a single study appropriately comparing the safety of mifepristone and acetaminophen or any of the common drugs presented in these claims, let alone a 20-year history of “clear and conclusive scientific evidence” to this end. Indeed, given that such a study would require similar indications for use, it is not possible for one to be undertaken. This alone is sufficient reason to qualify such comparisons as false and/or misleading and to contest their use to influence public and individual decision-making. To the extent that they still proliferate, however, it is also informative to examine the claims as they are presented. Such examination reveals the manifest deficiencies and disingenuousness of the assertions, in which statistics are presented that are neither comparable nor contextualized to fairly represent their limitations in assessing safety. What follows is an elaboration of these deficiencies, beginning first with a brief history of the claim.

## 3. The Origin of the Claim and Its Spread

Though the roughly two decades referenced by the Tong statement contain no scientific interrogation of this question, they do hold the origin of this line of argumentation. In a 2003 *Chicago Tribune* article detailing the death of a teenager after taking mifepristone, Dr. David Grimes, an abortion provider and past chief of the U.S. Centers for Disease Control and Prevention (CDC) abortion surveillance branch, was asked for comment [2]. Against the CDC’s then-current figure of slightly less than one per 100,000 deaths in those seeking abortion, he juxtaposed an uncited two per 100,000 death rate associated with penicillin and concluded that “having an abortion this way is safer than that”. Comparisons to sildenafil and acetaminophen soon followed in a 2004 editorial in the journal *Contraception*. There, authors from the Association of Reproductive Health Professionals and the University of California, San Francisco (UCSF) wrote that “…mifepristone is safer than Viagra”, again citing death rates, and that “acetaminophen (Tylenol) causes more deaths each year” [15].

These comparisons patterned the majority of those deployed in the following two decades: using death counts or death rates from different studies, at different times, and in vastly different populations, they asserted a reductionist definition of safety and compared numbers that are entirely incomparable. Paradoxically, the editorial itself provided ample ground for such criticism. Entitled “Presenting health risks honestly: mifepristone, a case in point”, it provided recommendations for the medical community to explain “objective health information…in a way that is clear, fair, and not misleading” [15]. But in comparing mifepristone to uncontextualized death rates and counts for sildenafil and acetaminophen, the latter of which was unsupported by its sources, the editorial violated three of its own recommendations: to compare drugs to their “reasonable alternatives”, to “put risk in context”, and to hold paramount “the importance of honesty”. These contradictions, as well as the violations of FDA guidance by all comparisons drawn between mifepristone and common drugs, will be explored below.

Irrespective of this unsteady foundation, the claim began to proliferate. Abortion provision organization Women on Waves [43] and research organization Gynuity Health Projects [46] picked up the comparison in the late 2000s, the former to penicillin and sildenafil and the latter to penicillin alone. Both resources presented the argument in Frequently Asked Questions sections, assuring women seeking abortion as well as abortion providers that concerns about its safety were unfounded. Interestingly, the comparison to acetaminophen largely laid dormant until 2016, when the group Advancing New Standards in Reproductive Health (ANSIRH) at UCSF picked up the claim published 12 years prior. In an issue brief on abortion restrictions, the authors repeated the comparisons and references offered in the *Contraception* editorial [22].

ANSIRH has since published three analyses, in 2019 [51], 2022 [52], and 2024 [21], in which the comparison is repeated. While a new reference is offered for the acetaminophen claim, these documents again provide an estimate of the number of yearly deaths associated with the drug in the United States, which, as in the case of the *Contraception* editorial, is not supported by its source. This number, in combination with the death rates attributed to penicillin and sildenafil, are the support offered for the conclusion repeated across all three documents that drug-induced abortion is safer than “using other common medications”. When claims of mifepristone’s superior safety to these three pharmaceuticals are cited, one of these ANSIRH documents is often the provided reference [3,7,8,12,13,23,42,53]. Included among these sources is Hey Jane, a telehealth abortion provider, which assures its clientele in state-specific resources that “the abortion pill is safer than Tylenol” and, later, Viagra, with both citing ANSIRH [42]. In another case, a magazine article linked to one of these analyses to support the statement that “[p]eer-reviewed research has shown that mifepristone is safer than Tylenol, which is widely available without a prescription” [12].

In some cases, however, it appears that the claim has reached such a consensus status that it need not be cited at all. Planned Parenthood’s topline text on its “*How safe is the abortion pill?*” page states that “it’s safer than many other medicines like penicillin, Tylenol, and Viagra” without citation [45]. In a particularly striking example, 12 major medical organizations co-signed an amicus brief in *Alliance for Hippocratic Medicine* (AHM) *v. FDA* that stated: “Mifepristone’s safety profile is on par with common painkillers like ibuprofen and acetaminophen, which more than 30 million Americans take in any given day” [29]. The in-line reference for this claim only supports the 30 million user figure; elsewhere in the brief, a footnote quotes a comparison drawn between mifepristone and NSAIDs, which will be addressed later in this work for its manifold deficiencies. Nowhere in the document is there a description of how a safety comparison to acetaminophen, or ibuprofen specifically amongst the broader class of NSAIDs, was drawn or sourced. As an ANSIRH source is cited elsewhere and for different purposes in the document, a plausible explanation is that the acetaminophen claim was internalized from that source and incorporated in a “summary” of mifepristone’s safety. The end result, however, is that an assertion is made with the appearance of common fact, not necessitating a direct reference for its substantiation.

That claims of this gravity, offered by organizations of such influence, should not be made without adequate reference, or any reference at all, is self-evident. Their continued advancement is evidence of the dangers of allowing this line of argumentation to proceed through the public square without the scrutiny that it demands. The claim comparing mifepristone to acetaminophen will provide the first case study in this scrutiny.

## 4. The Deficiencies of Reductive Comparisons

In recent ANSIRH documents, the reference provided for the comparison between mifepristone and acetaminophen is a study of FDA postmarketing data on painkillers from 2015 to 2016 [54]. Though this source notes 602 fatalities across the two-year timeframe studied, it is miscited to support the claim that “[a]cetaminophen…accounts for over 600 deaths annually” [21,51,52]. Prior to this, a separate source was cited in both the 2016 ANSIRH issue brief and the *Contraception* editorial that initiated the claim. Here, an investigation of the 308 liver failure cases observed across 41 months at 17 tertiary care centers noted that 120 were attributable to acetaminophen overdose, of which 87 survived [55]. The editorial used this source to state that “150 people are estimated to die each year in the US from acetaminophen-induced liver failure” [15], though the paper made no such attempt to extrapolate beyond its study of cases. ANSIRH, on the other hand, simply used the source to state that “medication abortion is safer than many common drugs in the US, including acetaminophen” [22].

These inaccuracies aside, the citation of these sources in no way meets the standard of providing “substantial evidence” for such comparative claims. Apart from this, their use can also be analyzed independently as a wholly inadequate representation of the safety considerations of either mifepristone or acetaminophen. Briefly, this concern arises from two considerations: the extent to which safety is rightly considered and the extent to which a reasonable basis of comparison is offered.

### 4.1. An Inadequate Consideration of Safety

In these sources, as in those cited in many similar statements, the only implied point of comparison is the death of the individual consuming the drug. Yet as described above, the FDA rightly considers far more than this in its assessments of safety. At the outset, it must be acknowledged that one such criteria is the effect of the drug “on the developing fetus” [48]; these comparisons drawn on mortality rates highlight the unignorable contradiction that a drug for any other indication that resulted in fetal death would be deemed unsafe, whether or not it was being compared with other drugs. Even with a sole focus on the individual consuming the drug, however, mortality alone fails as an adequate surrogate for drug safety. A few paragraphs removed from Dr. Grimes’s comparison to penicillin in the *Chicago Tribune* article, the author captures one reason why such a reductionist approach is inadequate:

“One problem with the drug…worries some mifepristone providers–it can be difficult for doctors to tell when a patient is truly in distress from the medicine. That’s because mifepristone, which is given along with the contraction-inducing drug misoprostol, routinely causes intense painful cramps and bleeding that last for five hours or more. The symptoms are similar to what might result from an infection or other life-threatening reactions” [2].

These concerns are prominently featured on mifepristone’s prescribing information in an FDA-mandated boxed warning—the “most well-known and serious” warning used by the FDA to communicate drug safety information on labeling [56]—that severe and life-threatening bleeding and infections can occur, the latter of which can defy typical presentation and be difficult to detect [57]. They are also addressed in the patient and prescriber agreements required as part of the Risk Evaluation and Mitigation Strategy (REMS) for mifepristone [58]. The possibility of these adverse events, compounded by the risk that they may be masked by commonly experienced effects of the drug, is an unignorable consideration regarding its safety.

It should also be noted that the description of side effects in the *Chicago Tribune* article is conservative. Mifepristone’s label states bleeding or spotting is expected for an average of 9–16 days, with up to 8% of patients bleeding for 30 days or more [57]. A small interview-based Norwegian study sought to relay anecdotes of women after drug-induced abortions: while some noted pain that was less intense than anticipated, others described pain that was “out of this world”, “much worse than giving birth”, and “a day in hell” [59]. These results were repeated in a recent study in which 1596 survey respondents described their pain experience after at-home drug-induced abortions [60]. On a ten-point scale, 41.5% of respondents rated their pain at least an eight; when asked to compare this pain to their anticipated experience, 30.4% selected that the pain was “a lot more than expected”. Yet because these side effects are known results of the drug’s mechanism of action, such experiences are dismissed despite both their severity and the possibility that they may mask an emergent condition. Indeed, after one patient in the Norwegian study “cried and screamed for eight hours”, she consulted the emergency department but was simply told that her experience was “quite normal”.

Because they are expected results, these side effects are also not listed in the table of reported adverse reactions on mifepristone’s label [57]. Explanatory text describing this omission has been echoed in public discussions, including a recent *New York Times* article that described papers cited in *AHM v. FDA* as “point[ing] to patient experiences that are common and expected, such as bleeding and pain” [61]. But these discussions also feature a similar dismissive approach to more serious complications such as surgical procedures to address incomplete abortions and emergency room visits, which were noted in preclinical studies to occur in 2.6–3.8% and 2.9–4.6% of abortions, respectively [57]. The *Times* article describes the former as “not typically dangerous” and the latter as “not a clear measure of serious complications”. A researcher at ANSIRH expressed the same, calling emergency room visits “not an indicator of abortion safety” [62]. Yet mifepristone’s boxed warning specifically mentions that an emergency room visit may be necessary should the patient experience symptoms such as “sustained fever, severe abdominal pain, [or] prolonged heavy bleeding” and be unable to reach a provided contact [57]. The patient agreement advises the same, warning that such symptoms “may be a sign of a serious infection or another problem” and “could require emergency care” [63].

Regardless of how one chooses to classify these events, it is entirely uncontroversial that they are critical considerations when evaluating and communicating the safety of a drug. Similarly, the concern expressed by mifepristone providers in the *Tribune* article, that commonly experienced effects of the drug can mask emergent complications, cannot be ignored. To brush aside these clinical realities and collapse all considerations of safety into a simple comparison of mortality is irresponsible and dangerous.

### 4.2. An Inconsistent Basis of Comparison

Beyond these concerns, even the statistics offered representing deaths in the comparator drugs are insufficient to make a valid comparison on that count alone. Though the sources were incorrectly cited, the implied comparison to the presented deaths per year associated with acetaminophen is the 32 deaths reported to the FDA as associated with mifepristone across a study period dating from 28 September 2000 to 31 December 2022 [64]. Presenting statistics in this fashion, however, ignores critical numerical and clinical context.

Numerically, a basic requirement of comparison is that raw numbers be normalized and not presented in isolation. But here we encounter one of many impossibilities in drawing direct comparisons between mifepristone and acetaminophen, that they are used by different populations, in a variety of dosages and forms (in the case of acetaminophen), and for vastly different purposes. To be clear, even if deaths per dose consumed were available as a metric, this would not enable an adequate comparison given that the users do not share a consistent basis across any of the above characteristics. This reality is presented plainly by the pharmaceutical advertising legal analysis in its notes that unrelated studies should not be juxtaposed side-by-side and that similar indications for use are required to make valid comparisons [50]. These are critical reasons why a well-controlled scientific investigation comparing the drugs has not been undertaken.

To the extent that raw death numbers are still cited as valid evidence for a safety claim, however, it cannot be ignored that these deaths occur within a vastly larger user base. Though the estimate of 30 million doses per day cited in the amicus brief above applies only to NSAIDs, acetaminophen use is also expansive. A 2006 study on the topic estimated that 19% of U.S. adults consumed acetaminophen per week; this translated to roughly 40 million Americans at that time [65]. Using another metric, 24.6 billion doses of acetaminophen-containing products were sold in 2008 alone [66]. While none of these numbers can be used to calculate a comparative mortality rate, they do provide an important perspective on the relative magnitude of acetaminophen use in the United States. In the 22-year timeframe noted above in which 32 mifepristone-associated deaths were reported to the FDA, there were an estimated 5.9 million mifepristone abortions [64].

More important than the numerical concerns with these claims, however, is the clinical context of the numbers referenced. In describing the patients with acetaminophen-induced liver failure, the study cited by the *Contraception* editorial and the 2016 ANSIRH brief specifically identified acetaminophen overdose as the cause of the observed liver failure, with a median dose of 13.2 grams per day consumed against the labeled maximum of four [55]. In this study, 36.67% of overdoses were described as intentional, 56.67% as accidental (occurring without intention for self-harm), and 6.67% unknown. A larger study of U.S. mortality and emergency databases found that intentional acetaminophen overdose accounted for between 55% and 74% of fatal cases, with 8–26% attributed to unintentional overdoses and 5–20% unable to be determined [67]. Of note, the authors of the former study wrote that “there is very little evidence of liver injury when acetaminophen is used according to package recommendations” [55].

Comparing the labeled use of one drug with the misuse of another in a safety claim is disingenuous. Not only do such constructs contradict the *Contraception* editorial recommendations noted above, they also directly satisfy yet another definition of a false or misleading claim by the FDA: an assertion that “presents information from a study in a way that implies that the study represents larger or more general experience with the drug than it actually does” [49]. More broadly, the inability to account for important numerical and clinical context in these comparisons is specifically identified by the FDA in its warnings against using postmarket data for this purpose [68].

## 5. Other Comparative Claims

As the other comparative claims involving mifepristone also cite only deaths or similarly reductionist metrics, the first element of scrutiny above, on the adequate consideration of safety, is broadly applicable. Regarding the second, on an inconsistent basis of comparison, the statistics offered in support of each individual comparison similarly break down under examination.

### 5.1. Penicillin

The originator of this style of claim, the comparison provided by Dr. Grimes, offers a penicillin death rate of two per 100,000 as its comparative metric. Though Grimes did not cite the statistic, subsequent publications reference a 2001 review article [69]. The authors of this work did not conduct the study behind this metric, and the citations offered on its behalf do not provide clear support for it. Following other citations in the review, however, and the publications that they in turn cite, it appears that the statistic of two per 100,000 may ultimately trace to a 1968 World Health Organization bulletin [70]. There, an author described a study he performed a year earlier: interviewing the 211 physicians in a Swiss town of approximately 200,000 people, he recorded four fatal anaphylactic reactions to penicillin. Later in the work, he rightfully cautions that “longitudinal community studies and epidemiological research on these phenomena are needed to illustrate their true frequency”.

Since then, improvements in the preparation and administration of penicillin, along with advances in our understanding and awareness of anaphylaxis, have drastically decreased the risk associated with the drug [71]. In an eight-year survey ending in 2017 across which nearly 6 million courses of penicillin were studied, 25 cases of anaphylaxis were reported with zero fatalities [72]. While the FDA specifically cites “favorable information about a drug…which [has] been rendered invalid by contrary and more credible recent information” as another example of a false and/or misleading claim [49], the persistence of this penicillin statistic despite more recent and rigorous evidence could be a reasonable corollary. Thus, claims made comparing mifepristone and penicillin both present a reductionist vision of safety and offer not only incomparable but also outdated statistics as their sole support.

### 5.2. Sildenafil

Most works claiming mifepristone’s superior safety relative to sildenafil cite a 2000 *JAMA* piece that discusses the 564 deaths reported across roughly 11 million patients prescribed Viagra in its first 1.5 years of approval [73]. While highlighting the need for further investigation, the article also notes the challenge of interpreting these deaths. Quoting the then-president elect of the International Society of Pharmacoepidemiology, the author writes that drugs like sildenafil are largely taken by individuals with preexisting complex diseases, making it difficult to disentangle the roles of the drug and the underlying condition(s) in the observed mortality. In fact, the condition for which sildenafil is primarily prescribed, erectile dysfunction, is a known indicator or precursor of cardiovascular disease and other associated comorbidities [74,75], a noteworthy link given that the described mortality was largely attributable to cardiac events. Still, the risks of taking sildenafil were investigated further in the following years; a synthesis of clinical trials and a prospective observational cohort study conducted from 2001 to 2004 found similar rates of both myocardial infarction and all-cause mortality in patients taking sildenafil versus placebo across over 1000 person-years of observation in each group [76].

Interestingly, ANSIRH’s recent analyses on mifepristone have ceased citing the 2000 *JAMA* article and now instead reference a study from 2010 on the adverse event reports associated with various phosphodiesterase-5 (PDE-5) inhibitors [77], of which sildenafil is a member. This paper does not offer the death rate ANSIRH claims; rather, it notes an unknown denominator of men using PDE-5 inhibitors, as well as the inability of its data source to establish causality. As will be described below, the FDA also advises against the calculation of such a rate using this data. Beyond the inability to provide a mortality rate, the source further makes known its inability to declare safety differences, even between members of this drug class, using its data. Another source on sildenafil safety makes a similar argument, noting that “it is not valid to compare the clinical efficacy and safety of [two other PDE-5 inhibitors] to sildenafil without the results of properly designed and conducted comparative studies” [78]. If members of the same drug class cannot be compared without a properly designed study, it is certainly the case that these works are in no way adequate support for a comparison with mifepristone.

This recent source aside, comparisons of drugs like sildenafil to mifepristone exemplify a key rationale underlying the caution against side-by-side comparisons of different studies. Here, the vast differences in those study populations’ respective risk factors cannot be properly accounted for when attempting to draw a safety comparison. Furthermore, as with the example of penicillin, both more recent evidence and the necessity to consider a more expansive definition of safety exacerbate the inappropriateness of this claim.

### 5.3. Nonsteroidal Anti-Inflammatory Drugs (NSAIDs)

The aforementioned amicus brief from major medical organizations in *AHM v. FDA* compared mifepristone to acetaminophen alongside ibuprofen [29]; aspirin is also occasionally claimed. The evidence the brief offers for the ibuprofen comparison presumably rests in a later-cited National Academies of Science, Engineering, and Medicine report, which concluded that “the reported risks [of drug-induced abortion] are both low and similar in magnitude to the reported risks of serious adverse effects of commonly used prescription and over-the-counter medications”, later clarified to include NSAIDs and antibiotics [79]. Unlike the sources described above, this report includes both death and some adverse events (AEs) in its comparison. Regarding NSAIDs, the report offers risk rates of hospitalization and death linked to NSAID use amongst elderly populations in the U.K. [80] and U.S. [81], which are then compared with the rates of significant AEs (“hospital admission, surgery, blood transfusion, emergency department treatment, or death”) and deaths associated with mifepristone use recorded in a 2017 study on telemedicine abortion [82].

The first concern with these statistics, like those presented by the sildenafil comparison, is at the level of population. The report admits that the cited morbidity and mortality risks are assessed only in elderly patients. To give this admission more context, another study estimating mortality from NSAID-associated gastrointestinal events noted that the mean age in fatality cases was 76 years and that 89.7% of such fatalities occurred in those over 60 years of age [83]. The same study noted that approximately half had a history of gastrointestinal disease and that nearly 57% of deaths were not due to the event itself but rather were “a consequence of complications developed during hospitalization prompted by the gastrointestinal event”, an occurrence more likely in the population in which the statistic is being assessed. Again, we encounter the impossibility of comparing two entirely disparate populations, particularly those with different risk factors for morbidity and mortality.

The second concern exemplifies another rationale against combining unrelated studies in a comparison: differences in protocol. Investigation of the statistics used to represent risk rates for NSAIDs reveals that they are not even internally consistent within the report, let alone comparable to separate studies of mifepristone deaths and AEs. One presented metric, a 0.23% risk of hospitalization amongst elderly U.K. residents, was calculated by extrapolating a study of gastrointestinal bleed hospitalizations owing to NSAID exposure to the general population level and then dividing by the estimate of U.K. elderly who take NSAIDs [80]. The risk rate for mortality was constructed in a similar manner. On the other hand, the cited “even higher” hospitalization risk rate of 1.25% in the U.S. took hospitalizations observed in an elderly Tennessee Medicaid cohort from 1984 to 1986 and normalized to person-years of study [81]. After subtracting out baseline hospitalizations, the authors found an excess rate of 12.5 per 1000 person-years. These numbers are not comparable to each other, let alone to the serious AE and death rates presented in a 2017 prospective cohort study on telemedicine abortion.

The support provided for the claim that mifepristone is safer than, or as safe as, NSAIDs perfectly encapsulates the dangers of juxtaposing unrelated studies. These reports, taken from different time periods, assessing different populations, and using different methods, cannot be reasonably compared.

### 5.4. Claims Made Between Multiple Drugs on Adverse Event Rates

While the National Academies report cited a study that assessed its own serious AE rates for telehealth abortion, other claims that incorporate AEs into their safety discussions typically cite one of two sources for mifepristone or its comparators: premarket data described in the drug label and postmarket data presented through the FDA’s AE reporting infrastructure. The utilization of any AE data, whether from these sources or outside them, for a comparison exacerbates the dangers of combining unrelated studies; while the concerns about different conditions, populations, and indications for use continue to apply, AE data also suffers from inconsistent definitions and incomplete reporting. These realities are acknowledged by the FDA in explicit warnings against using such data for the purposes of comparison.

Premarket AE data, communicated under the corresponding section on a drug’s label, is recommended by FDA guidance to be preceded by the following statement: “Because clinical trials are conducted under widely varying conditions, adverse reaction rates observed in the clinical trials of a drug cannot be directly compared to rates in the clinical trials of another drug and may not reflect the rates observed in practice” [84]. This statement makes clear that even drugs within the same class and with the same indication for use should not be compared in this manner. Similar cautions are applied to postmarket data, as described above, with the FDA noting that this data “cannot be used to calculate the incidence of an adverse event or medication error in the U.S. population” and that it “should not be used to determine the safety profile of one drug compared to another” [68]. These cautions have many origins, including the inability of AE reports to establish causality, an unknown denominator of those taking the drug, and the passive reporting structure of AEs in which medical professionals and/or consumers voluntarily report experiences to the FDA. Though the mifepristone prescriber agreement originally supplemented this process with a requirement that prescribers report serious AEs (death, life-threatening complication, or hospitalization) of which they became aware [85], even these have been noted to be severely undercounted, attributable to both failures in the mandatory chain [86] and management of abortion complications outside of that chain [87].

Despite these warnings and realities, several sources still leverage this data and, in doing so, provide case studies in these dangers. One such resource is a document by NARAL Pro-Choice America (now Reproductive Freedom for All) entitled “Mifepristone is a Safe Choice”, in which mifepristone and 15 unrelated drugs are compared on AE rates [88]. Each is presented favorably against a cited mifepristone rate of 0.28%, a striking juxtaposition for many of the drugs listed (e.g., Advair Diskus, an asthma medication with a listed rate of “at least 27%”). The rates for the 15 comparator drugs are cited to premarket data, which alone demonstrates the tenuousness of such a construct: according to the respective labels, the 27% of Advair users who experienced an upper respiratory tract infection [89], for example, are compared with the 13.9% of Xanax users who experienced depression [90]. These numbers are reported independently of their placebo baselines (14% and, importantly, 18.1%, respectively) and are, as the authors note, sometimes selectively presented: “some particularly high outliers” are omitted, as are “some especially common side effects”. This caveat, however, is not the explanation for the exclusion of all seven AEs listed on the same section of mifepristone’s label with rates between 18 and 75%. Instead, this exclusion traces to the presentation of postmarketing data for mifepristone, a choice unique amongst the comparator drugs. The cited source for the 0.28% rate, an FDA summary of AE reports, only presents raw counts of reports received across various AE categories and an estimate of 1.52 million mifepristone abortions in the same timeframe [91]. Interestingly, a rate of 0.28% cannot be calculated from any of this data; yet the far more critical point is that the data cannot in any way be used in the calculation of such a percentage. Its deployment, and its use against already ill-advised comparisons between premarket AE rates in other drugs, is disingenuous and, again, dangerous. The effects of colocalizing these incomparable percentages in a table were reflected in a peer-reviewed academic article, which cited the NARAL resource to state that “in the 17 years since mifepristone has been approved in the United States, there have been far fewer adverse events than from either acetaminophen or fluticasone [Advair]” [16].

The NARAL source is not alone in improperly describing AEs attributable to mifepristone in the service of a comparison. A model resolution provided by the Public Leadership Institute [92] and introduced in Missouri [93] and Wisconsin [94] claims that “medication abortion causes no complications in more than 99.9 percent of cases, making it safer than Tylenol, aspirin and Viagra”. Another example is found in a resolution adopted by the American Academy of Family Physicians (AAFP) College of Delegates, which notes a “0.05% risk of major complications” for mifepristone and an unspecified “higher complication rate” for “acetaminophen, aspirin, loratadine, and sildenafil” [95]. Online resources for the former reference a Guttmacher Institute Policy Report [96], which does not offer such a percentage. It does, however, cite the same FDA postmarket summary as the NARAL resource in claiming that “of the 1.52 million women in the United States who used Mifeprex between 2000 and 2011, 612 were hospitalized”. Despite the inappropriateness of using this source, and of treating incomplete hospitalization records as representative of complications, this statistic is on record in two statehouses and on offer to others. The latter claim is of particular note given its adoption by a major medical society; it is also among the sources offered for the comparison in the attorneys general’s motion introduced at the outset of this work [24]. Here, rather than citing sources incorrectly, the AAFP resolution simply cites incorrect sources: though one version of the document contains no citation, another references a study on aspiration abortions that makes no mention of mifepristone [97].

Though most comparisons drawn between mifepristone and other drugs deal solely with mortality in those consuming the drug, those that introduce AE rates demonstrate the particular dangers that can befall such an approach. The FDA is clear that such data, whether from clinical trials or postmarketing surveillance, should not be used for such a purpose. Beyond this, the examples above also display the profound risks of selectively or incorrectly presented data leveraged in these lines of argumentation.

It must finally be noted that future similar assertions, should they continue to be made, will rest on data that will be even more inadequate to assess safety. Changes in mifepristone regulations in 2016 [98], 2021 [99], and 2023 [58] collectively repealed the prescribers’ requirements to report serious adverse events besides death and eliminated all requirements for in-person visits. Recent years have also witnessed the rise of “self-managed abortions” in which pills are obtained and used without any contact in the U.S. healthcare system [1]. Though the FDA recommends against this practice [100] and has issued warning letters on the topic [101,102], the practice continues: a study surveying such sources across the U.S. found an average of 5931 “provisions per month” between July and December of 2022 [103]. With this method, the reporting chain to the FDA is definitively broken so that even deaths resulting from mifepristone may be unknown. Emerging trends in the counseling of patients that disclosure of their abortion is unnecessary when presenting with complications [5,104,105], as well as the recent recommendation by the American College of Obstetricians and Gynecologists to avoid reporting outcomes of self-managed abortion unless legally compelled [106], further limit the identification of AEs, including deaths, associated with mifepristone. Collectively, these developments have further lessened the already limited ability of postmarket reports to inform reliable discussions of mifepristone’s safety, let alone to be deployed in already unadvised comparisons.

## 6. Conclusions

The reductive claim that “mifepristone is safer than Tylenol” and the class of similar comparisons that it represents are wholly inadequate distillations that attempt to inform a topic of critical public concern. That the FDA would demand a controlled head-to-head study to support such a claim by the manufacturer is informative; that one has never been undertaken is similarly illuminating. Put simply, it is not possible to draw any conclusion from the comparison of drugs with different uses, administered in different manners, and used by individuals with different risk factors.

Not only have the comparisons between mifepristone and other drugs failed in their duty to adequately assess this impossibility, but they have also demonstrated a complete disregard for the need to communicate comprehensive and truthful safety information to patients, policymakers, jurists, and the public. In collapsing complex safety considerations into simplistic comparisons that leverage wholly incomparable metrics, these assertions systematically violate the norms and regulations that inform evidence-based biomedical communication. Despite this, however, they have reached both the most diffuse and influential levels of our discourse over the span of roughly two decades, buoyed by the false and dangerous perception of scientific reference and expert consensus.

Thus, while a primary offering of this work is a direct counterpoint to these claims, their proliferation also merits broader consideration. In these case studies, assertions meant to decrease regulations and quell public concerns about a drug advanced without the scrutiny that should have been demanded of them. As demonstrated in this work, the interrogation of source material and comparison to established norms are effective and necessary tools to this end. Indeed, in this case, exploring cited references served as a particular validation of those norms as warnings and guardrails against the multiplicity of errors that can befall any unfounded safety comparison drawn between drugs. Finally, the role of large groups, most notably medical societies, in the proliferation of these claims cannot be understated. In a particular way, this analysis offers a call for awareness of the memberships on whose behalf these claims are made, who in this instance were presented as a unified body of experts supporting an untenable consensus.

The pace at which these claims have proliferated, the breadth of venues in which they are offered, and the now growing assignment of them to the realm of common fact collectively demonstrate the profound ramifications of failing to identify them as what they are: unfounded, misleading, and entirely dangerous assertions. We can and should demand more of those who present drug safety data in this manner and oppose the use of these claims in informing regulation, policy, and patient decision-making.

## Data Availability

No new data were created or analyzed in this study.

## Abbreviations

The following abbreviations are used in this manuscript:

AAFP	American Academy of Family Physicians
AE	Adverse Event
AHM	Alliance for Hippocratic Medicine
ANSIRH	Advancing New Standards in Reproductive Health
CDC	U.S. Centers for Disease Control and Prevention
FDA	U.S. Food and Drug Administration
NSAID	Non-Steroidal Anti-Inflammatory Drug
PDE-5	Phosphodiesterase-5
REMS	Risk Evaluation and Mitigation System
UCSF	University of California, San Francisco
